# Bacterial and viral infections and related inflammatory responses in chronic obstructive pulmonary disease

**DOI:** 10.1080/07853890.2020.1831050

**Published:** 2020-11-04

**Authors:** Silvestro Ennio D’Anna, Mauro Maniscalco, Francesco Cappello, Mauro Carone, Andrea Motta, Bruno Balbi, Fabio L. M. Ricciardolo, Gaetano Caramori, Antonino Di Stefano

**Affiliations:** a Divisione di Pneumologia, Istituti Clinici Scientifici Maugeri, IRCCS, Telese, Italy; b Dipartimento di Biomedicina, Neuroscienze e Diagnostica avanzata (BIND), Istituto di Anatomia Umana e Istologia Università degli Studi di Palermo, Palermo, Italy; c Euro-Mediterranean Institute of Science and Technology (IEMEST), Palermo, Italy; d UOC Pulmonology and Pulmonary Rehabilitation, Istituti Clinici Scientifici Maugeri, IRCCS di Bari, Bari, Italy; e Institute of Biomolecular Chemistry, National Research Council, Pozzuoli, Italy; f Divisione di Pneumologia e Laboratorio di Citoimmunopatologia dell’Apparato Cardio Respiratorio, Istituti Clinici Scientifici Maugeri, IRCCS, Veruno, Italy; g Dipartimento di Scienze Cliniche e Biologiche, Università di Torino, AOU San Luigi Gonzaga, Torino, Italy; h Pneumologia, Dipartimento di Scienze Biomediche, Odontoiatriche e delle Immagini morfologiche e funzionali (BIOMORF), Università degli studi di Messina, Italy

**Keywords:** T-lymphocytes, ILCs, B cells, NK cells, macrophages, dendritic cells, NETosis, autophagy, pyroptosis, autoimmunity, outcome, disability

## Abstract

In chronic obstructive pulmonary disease (COPD) patients, bacterial and viral infections play a relevant role in worsening lung function and, therefore, favour disease progression. The inflammatory response to lung infections may become a specific indication of the bacterial and viral infections. We here review data on the bacterial–viral infections and related airways and lung parenchyma inflammation in stable and exacerbated COPD, focussing our attention on the prevalent molecular pathways in these different clinical conditions. The roles of macrophages, autophagy and NETosis are also briefly discussed in the context of lung infections in COPD. Controlling their combined response may restore a balanced lung homeostasis, reducing the risk of lung function decline.

**KEY MESSAGE**
- Bacteria and viruses can influence the responses of the innate and adaptive immune system in the lung of chronic obstructive pulmonary disease (COPD) patients.
- The relationship between viruses and bacterial colonization, and the consequences of the imbalance of these components can modulate the inflammatory state of the COPD lung.
- The complex actions involving immune trigger cells, which activate innate and cell-mediated inflammatory responses, could be responsible for the clinical consequences of irreversible airflow limitation, lung remodelling and emphysema in COPD patients.

## Introduction

The respiratory system is populated by several species of bacteria and viruses, which establish a complex system of mutual relationships among them and with the host. Compared to healthy controls, chronic obstructive pulmonary disease (COPD) airways show higher levels of inflammation, which is the cause of the disease progression [1,2].

In stable COPD patients and during exacerbation, the pulmonary microbiota changes its composition, and keeps changing during disease progression. Due to the alterations in quantity and functioning of cells in the COPD immune system, viruses and bacteria could present a different pathogenicity [3], and their interaction with the COPD respiratory system is a major cause of exacerbations and may amplify chronic inflammation in stable COPD [4]. Acute exacerbations in COPD are associated with higher mortality [4,5] because of the possible reduction of lung function. They can last several weeks and increase disease severity by accelerating the rate of lung function decline [6].

The inflammatory process, the oxidative stress and bacteria/viruses colonizing and/or infecting the airways, play a fundamental role in airflow worsening and disease manifestations. Small airways remodelling and pulmonary emphysema are most likely the results of the chronic inflammation response to inhaled xenobiotics [7]. While in COPD patients, the pattern of cellular prevalence in the small airways and parenchyma is well defined [2], more data are required to better understand the patterns of lung inflammation and immune response in the different COPD phenotypes, and the interactions with the microbiota.

In this review, we will discuss the interaction of bacteria and viruses with the host immune response in the respiratory system, and the response of the inflammatory cells in COPD patients.

## Bacteria and viruses in COPD

### Bacteria in stable COPD

Until the development of non-culture-based methods (i.e. quantitative polymerase chain reaction, qPCR), the lower airways of healthy individuals were considered an almost sterile environment. That an ecosystem of microorganisms could colonize the lungs like the gastrointestinal tract was an uncommon idea [8], mainly because the study of lung-resident microorganisms by culture techniques is difficult. Possible limitations, among others, are the likely contamination of samples, the relatively small number of bacteria present in the lower airways, the impossibility to culture ca. 70% of bacteria by current techniques, and the difficulty to culture many species in the remaining 30% [9,10]. Application of qPCR techniques has shown that the lungs of healthy individuals are colonized by a wide spectrum of bacteria [11], and that a different combination of bacterial species affects the lungs of COPD patients, with respect to healthy individuals [12]. The predominant species isolated in COPD lower airways are *Haemophilus influenzae*, *Streptococcus pneumoniae*, *Moraxella catarrhalis* and *Pseudomonas aeruginosa* [13,14] in those with a severe disease.

The total number of bacteria and the microbiota composition in COPD airways correlate with inflammation [8]. In fact, using sputum cultures and qPCR, a positive correlation between the airways’ bacterial load and the bronchial inflammation has been reported [15]. In particular, the number of *M. catarrhalis*, *H. influenzae*, *S. pneumoniae* and the airways’ inflammation are strongly correlated [16]. Barker et al. [17] demonstrated that the detection of bacteria in the airways of stable COPD correlated with pro-inflammatory cytokines secreted in the sputum (IL-1β, IL-10 and tumour necrosis factor (TNF)-α) and decreased CCL13. In dendritic cells (DCs), common pathogenic bacteria of the airways such as *Haemophilus* and *Moraxella*, can stimulate 3–5 times more efficiently the secretion of IL-10, IL-12p70 and IL-23, as compared with commensal gram-positive bacteria like *Actinomyces* [18]. In DCs, *Prevotella* species are able to reduce by ca. 50% the secretion of IL-12p70 induced by *Haemophilus* [18]. It was hypothesized that, as in the gut, bacteria are able to modulate the immune response to pathogens (e.g. the *Haemophilus*), helping the host to clear them from the airways. Most likely, gram-negative bacteria and lipopolysaccharide induce the production of specific cytokines, which stimulate components of the innate immunity system and the Toll-like receptor 4 (TLR4)-mediated inflammatory response [18]. This has been partially confirmed in bronchial biopsies of stable severe COPD patients, where a direct relationship between *P. aeruginosa* bacterial load, bronchial inflammation and overexpression of TLR4 has been found [19]. An increased number of lung *Lactobacillus* has been described for COPD patients, and it was suggested that they could act as a target of inflammation or an immune modulator of the inflammatory response [20].

The microbiota composition could also favour disease progression. In the bronchoalveolar lavage (BAL) of smokers with normal lung function, Erb-Downward et al. [21] observed a reduced variety of microbiota with respect to healthy individuals. They hypothesized that the relative diversity reduction could be persistent and could be either an *effect* of the lung inflammation or, in part, a *cause* of disease onset and progression. Reduction of bacterial variety was reported in sputum of stable COPD, particularly in patients with more severe disease, hypothesizing a substitution of bacterial flora for species that are only marginally present in patients with a less severe disease [22]. Such macrobiota alteration could induce further lung inflammation and worsen the disease [22]. In severe COPD, a microbial variation with a relative increase of *Proteobacteria* and *Actinobacteria*, and a reduction of *Firmicutes phyla* was reported [23]. In parallel, a significant association of CD4^+^ cells with the extent of emphysema and bronchial inflammation was also found [23].

Evidence has been reported that reduction in diversity and richness of the microbiota is correlated with greater emphysema and immune cell infiltration in lung tissue [23,24]. Differences in microbiota composition have been observed in patients with different lung alterations detected by CT scan [25]. Bacteria can also change the host environment by increasing the viral pathogenicity, and therefore worsening the bronchial inflammation. *H. influenzae* can favour rhinovirus (RV) infection by enhancing the expression of intercellular adhesion molecule-1 (ICAM-1) in the respiratory epithelium, which can be used by the respiratory viruses to bind and invade the target cells [26]. In an *in vitro* model, proteases from bacteria commensal in the airways could enhance the pathogenicity of influenza virus by cleaving their haemagglutinin and favouring the endocytosis from the target cells [27].

Bacteria interact with the adaptive and innate immune system. They alter the environment and compete with each other for nutrients and space by producing substances capable of inhibiting or increasing the growth of a different species [27]. Interestingly, Valdez et al. [28], in an *in vitro* study and in a murine model of burns infected with *Pseudomonas*, showed that cultures and filtrates of *Lactobacillus plantarum* were able to reduce the pathogenicity of *P. aeruginosa* by reducing the secretion of elastase and biofilm production [28]. Accordingly, three weeks of daily administration of *Lactobacillus casei* in male smokers increased the cytotoxic activity of natural killer (NK) cells that was reduced by cigarette smoke [29]. Pathogen-free mice colonized with *Staphylococcus aureus* normally resident in the airways of wild mice showed a reduced mortality when infected with influenza virus [30].

**Figure 1.**
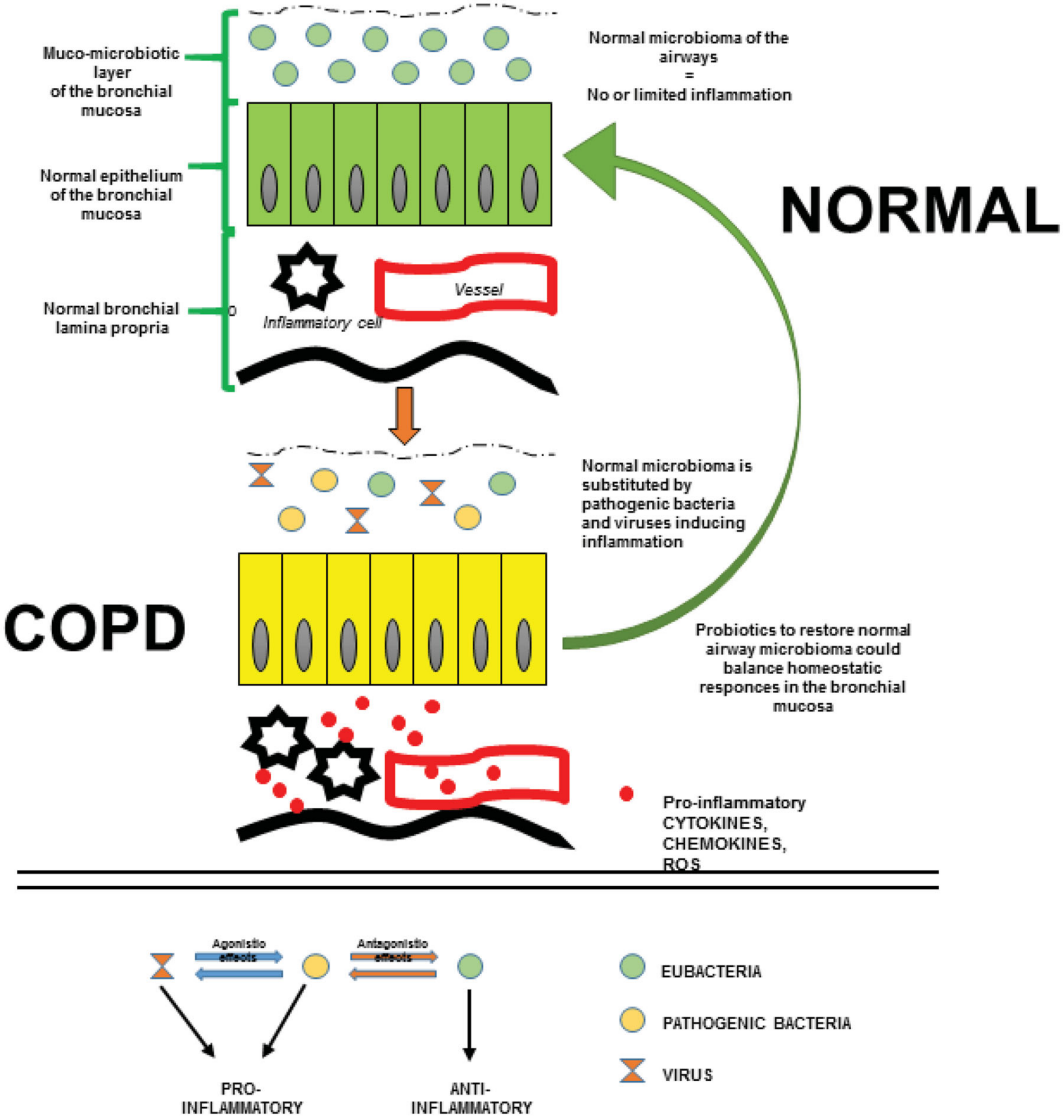
Schematic representation of the changes in microbiota and inflammation in COPD patients. Top, airway mucosa of healthy subjects presents a normal muco-microbiotic layer characterized by eubacteria. In contrast, in COPD patients, the muco-microbiotic layer is altered by pathogenic bacteria and viruses, which are associated with an inflammatory state of the bronchial-bronchiolar mucosa (epithelium and lamina propria). As a hypothesis, administration of probiotics may ameliorate the histopathological features of the airways, contributing to restoring a normal homeostasis in the lung. Bottom, the scheme shows that: (1) eubacteria have anti-inflammatory effects; (2) pathogenetic bacteria and viruses have pro-inflammatory effects; (3) eubacteria and pathogenic bacteria show antagonist actions; (4) pathogenic bacteria and viruses show agonist-challenging and pro-inflammatory actions.

According to the above data, selected bacterial species could reduce both the bacterial pathogenicity and the inflammation in the airways of stable COPD. This could be more than a working hypothesis since progresses in the knowledge of lung microbiota could prompt an understanding of the relationship between microbiota and COPD clinical phenotype (Figure 1).

### Bacteria in COPD during exacerbations

COPD exacerbations are associated with changes in airway microbiota and airway inflammation. Bacteria are isolated in nearly 50% of the total COPD exacerbations as shown by a meta-analysis on 118 studies, which included over 83% culture-based isolation methods [31]. The isolation of new strains of *H. influenzae*, *M. catarrhalis* or *S. pneumoniae* in sputum was linked to a considerably increased possibility of exacerbations [32]. In fact, COPD patients experiencing an exacerbation have a high probability of developing lung dysbiosis, particularly those who are frequent exacerbators [33]. Changes in microbiota can discriminate among different types of exacerbations of different aetiology. Wang et al. [34] observed a wider difference in microbiota changes considering bacterial vs. eosinophilic exacerbations. During bacterial exacerbations, they reported a decrease in *Streptococcus* and an increase in *Haemophilus*, while eosinophilic exacerbations induced a decrease of the *Proteobacteria*/*Firmicute*s ratio [34]. A COPD subgroup with high *Gammaproteobacteria/Firmicutes* ratio in sputum samples was found during an exacerbation, and it strongly correlated with FEV_1_ reduction and increased inflammatory markers [35,36]. A possible reason was that *Proteobacteria* are a major phylum of gram-negative bacteria, including *Pseudomonas*, *Acinetobacter*, *Haemophilus* and *Moraxella*, which could increase the level of lung inflammation. On the contrary, *Firmicutes*, represented by gram-positive bacteria, including *Streptococci* and *Lactobacilli*, did not show a similar pro-inflammatory action [36]. Furthermore, the subgroup with a high *Gammaproteobacteria/Firmicutes* ratio benefitted from antibiotic therapy with respect to other subgroups of patients with a high *Firmicutes/Gammaproteobacteria* ratio or with a balanced *Gammaproteobacteria/Firmicutes* ratio, capable of maintaining a more stable bacterial population of the lung even during an exacerbation period [36].

More recently, the sputum of GOLD 2 and 3 COPD patients, during stable state and six days after the onset of an exacerbation, confirmed a microbiota shift related to an increase of both *Firmicutes* and *Proteobacteria* [35]. Wilkinson et al. [37] confirmed an increased risk of exacerbation associated with the acquisition of a new bacteria strain like *Moraxella catarrhalis* in the airways of COPD patients. Interestingly, the risk of exacerbation driven by non-typable *Haemophilus* was correlated with the winter season, suggesting a possible correlation between non-typable *Haemophilus* and *Human rhinovirus* (HRV) infection [37].

**Figure 2.**
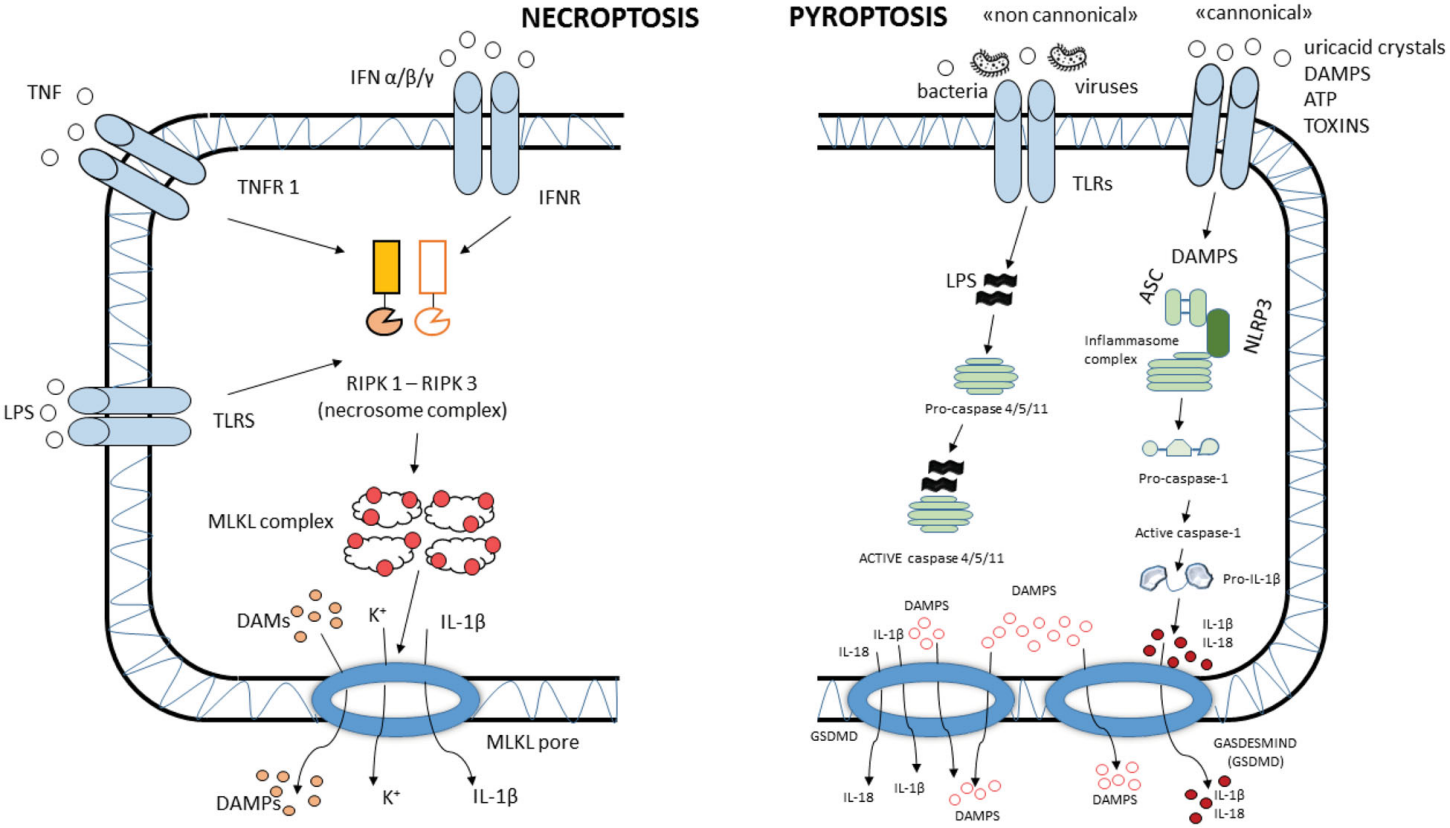
Schematic representation of necroptosis and pyroptosis cellular functions. In necroptosis, pro-inflammatory cytokines and/or LPS bind their own receptors on the cell membrane, which activate the necrosome complex. This, in turn, activates the MLKL complex damaging the cellular membrane, MLKL pore formation and release of DAMPS, K^+^ and IL-1β. In the pyroptosis “canonical model”, DAMPS, toxins, ATP, uric-acid crystals or other cellular stressors activate the inflammasome complex, which can bind pro-caspase 1 involved in the activation of caspase-1 followed by activation of IL-1β and IL-18 that are released through gasdermin pores. In the “non-canonical” pyroptosis model, bacteria, viruses and other stressors bind procaspase 4/5/11, which, after activation, induces the production and release through gasdermin pores of DAMPS and proinflammatory cytokines such as IL-1β and IL-18. DAMPs: damage-associated molecular patterns; TLR: toll like receptor; GSDMD: gasdermin D; MLKL: mixed lineage kinase domain like pseudokinase; RIPK1-3: receptor-interacting protein kinase 1–3.

Recent studies have observed how some pathogenic bacteria like *Klebsiella pneumoniae* increase inflammation in the airways inducing necroptosis [38]. Moreover, toxins produced by bacteria such as *S. aureus*, *S. pneumoniae* and *Serratia marcescens*, can also induce necroptosis [39]. The extreme effectiveness of these toxins on immune cells (neutrophils, T cells and macrophages) can dysregulate the immune response by reducing the number of immune-regulatory cells and eventually increasing the level of airways’ inflammation [40] (Figure 2). Presently, there is no grading of the exacerbation severity, making more difficult the identification of selected COPD phenotypes. Classification of the exacerbations related to COPD severity would be helpful to better define the role of the microbiota with respect to the COPD disease state [41]. Few data are available regarding the long-term effects of antibiotics and steroids in influencing the composition of the lung microbiota. Wang et al. [34] observed a prolonged, undifferentiated effect of oral steroids and antibiotics, the steroids decreasing alpha diversity with an increase of *Haemophilus* and *Moraxella* [34]. The effect on the microbiota due to the treatment lasted for a long period, until the patient complete recovery [34].

### Viruses in COPD in stable state

The development of qPCR allowed the observation of a viral community in the lower airways in stable COPD patients. Seemungal et al. [42] reported the presence of viruses in nasal aspirates and blood samples of stable COPD patients. In particular, the authors found RVs, coronavirus, parainfluenza virus and chlamydia in over 16% of the samples collected from 68 patients. Among all viruses, the RSV was the most present (27.5% of the samples). Papi et al. [43] found virus persistence in a considerably less proportion of enrolled patients. They found the persistence of respiratory viruses such as RSV and RVs in only 6.2% of sputum samples from 64 stable convalescence COPD patients. Wilkinson et al. [44], in a longitudinal study, observed the persistence of RSV in 79.7% of the 74 enrolled patients, and proved that the persistence of RSV in COPD airways was linked to higher inflammation and respiratory function decline, while McManus et al. [45] isolated viruses from sputum samples in 11.8% of 68 stable COPD patients. The presence of viruses, namely, influenza virus A and coronavirus, was also confirmed in lung tissue of stable COPD patients who underwent surgery for lung cancer. The authors reported a direct relationship between the presence of inflammatory cells and total viral load [46].

Matsuse et al. [47] evidenced the presence of adenoviral E1A protein in COPD airways, and hypothesized the possibility of a latent viral infection in those patients. In animal models with an adenovirus latent infection, an increased inflammatory response was observed after acute exposition to cigarette smoke and a higher extent of emphysema after chronic smoke exposition [48]. Using an *in vitro* model, evidence was reported that E1A adenovirus protein could worsen the inflammation in COPD via NF-kB by inducing the expression of ICAM 1 and upregulating the production of IL-8 on the airway cells surface [49]. These *in vitro* findings were confirmed on lung tissue by the evidence that the expression of adenoviral proteins in alveolar epithelial cells correlates positively with the extent of lung destruction in patients with emphysema [50].

The Epstein-Barr virus (EBV) and the cytomegalovirus (CMV) have been found in the airways of COPD patients. In fact, Polosukhin et al. [51] reported that EBV prevalence positively correlated with the severity of the disease and the degree of inflammation. McManus et al. [52] described the presence of EBV in stable and in acute COPD states, as the virus could persist in the airway epithelium. There is *in vitro* evidence that EBV-latent membrane protein-1 increases inflammation by inducing ICAM-1 expression in the airway epithelial cells via NF-kB pathway [53]. It is not clear if the presence of these herpesviruses increases the airways’ inflammation, or if the alterations of local immune system (which can occur with the progression of the disease) associated with the use of steroids, could cause the increased presence of the viruses in the airways. Moreover, although the presence of viruses in the airways of stable COPD is well documented [54], a highly variable percentage, ranging between 6.25% [43] and 79.7% [44], has been reported. Such variability might depend on the use of medications like steroids, disease severity and the collection time of the samples, among other factors. Other possible explanations could be the lack of susceptibility of some groups of COPD patients to viral infections, and the persistence of viruses in the airways [55].

The viral load of the most common viruses populating the COPD airways was recently quantified. In patients without exacerbations for at least 6 months and not using oral or inhaled corticosteroids for one month, the total viral load in the large bronchial rings and in the lung parenchyma was similar in mild/moderate COPD and control smokers with normal lung function. This finding was associated to relatively high levels of viral-related markers in tissue specimens from large airways and lung parenchyma, suggesting a “primed” state of the bronchial mucosa in those patients [56]. These data indicate that clinical conditions of the patients is mandatory and should be clearly defined when studying viral load and related inflammatory response in COPD patients.

Bacteriophages, viruses infecting prokaryotic cells, are another fundamental component of the viral community. By infecting pathogenic bacteria, these microorganisms can carry and spread interspecies antibiotic resistance and virulence genes, favouring airways infection by antibiotic resistant bacteria [57] and chronic infections [58]. As an indirect support, some bacteriophages, when present in infected wounds in humans and animal models, could worsen the infection, triggering antiviral immunity of the host and reducing the clearance of the bacteria infecting the lesion [59]. The same action can be hypothesized for bacteriophages in the airways of stable COPD patients, where the presence of viruses is related to a higher degree of inflammation and a lower level of respiratory function [52,60]. The role of bacteriophages in modulating bacterial–viral infections and related inflammation, however, requires further investigations. Hypothetically, the development of new treatments that could reduce the viral load may be useful to reduce airway inflammation and may be helpful to control the bacterial infections. Treatments based on phages could also be developed to target some pathogenic bacteria [61]. Alternatively, viruses infecting prokaryotic cells could be used to vehiculate into pathogenic bacteria toxins or genes that could reduce their pathogenicity by controlling, for example, antibiotic resistant bacteria [62,63].

### Viruses during COPD exacerbations

The PCR development has better defined the role of viruses as trigger of COPD exacerbations. Two meta-analyses [64,65] showed the prevalence of viruses in about 40% of cases. Jafarinejad et al. [65] examined 28 studies (including 1304 patients) finding a virus occurrence in 43% of COPD exacerbations, identifying mainly RV, influenza A and RSV but also metapneumovirus, coronavirus, adenovirus and parainfluenza viruses [65,66]. Similar results were observed for 127 patients followed longitudinally [37]. In hospitalized patients, the presence of respiratory viruses was observed in 29.2% of nasopharyngeal swabs, bronchial aspirates and BAL specimens. Moreover, 60.2% of all identified viruses were picornaviruses (RV or enterovirus) and influenza viruses [67]. The presence of viruses in COPD airways varies from ca. 60% [42] to roughly 20% [68]. Such a wide variability could be explained by geographic differences or prevalence of frequent exacerbators in the study population, the collecting time at different points of the biological specimens at the onset of the symptoms, the techniques of virus isolation [69], among others variables.

COPD exacerbations sustained by viruses and virus–bacteria coinfections seem to be more severe with respect to the bacterial ones. Some viruses like RV, can cause severe symptoms together with a reduction of lung function, which last for several weeks [8]. This is a consequence of the fact that in the airways of COPD patients, RV can stimulate the production of proinflammatory cytokines (e.g. IL-8) more than in healthy controls [70]. Seemungal et al. [42] observed for patients, who experienced a viral exacerbation, a longer median daily total symptom count recovery time, a higher total daily symptom count, and a higher frequency of exacerbations in comparison with those who experienced a non-viral exacerbation. The authors isolated viruses in 64% of the 83 exacerbated patients, and observed a coinfection with RV and other respiratory viruses (coronavirus, RSV and influenza virus) in 6.5% of the patients.

McManus et al. [45] observed an increased disease severity in patients with a viral coinfection, while Ko et al. [68] reported an increased severity for patients with a positive nasopharyngeal aspirate PCR and a viral culture with respect to patients with positivity only in nasopharyngeal aspirate PCR. This could be explained by the fact that the positivity in viral culture indicates active and replicating viruses. In fact, a PCR positive for viruses only reveals the existence of DNA/RNA, but does not demonstrate the presence of viral replication inducing exacerbation, unless the viral load in the airways is determined [71].

In an *in vivo* model of rhinoviral infection in moderate COPD patients, Mallia et al. [72] demonstrated the capability of RV to induce exacerbations in COPD after inoculation in the airways. Moreover, the authors reported that the amount of viruses correlated with markers of inflammation and reduction of histone deacetilase-2 activity, and that elimination of the viruses by the immune system preceded the resolution of the symptoms [73].

Some coronaviruses, on the other hand, seem to be more efficient than other common respiratory viruses to invade the lower respiratory tract [74] and cause a viral pneumonia [75]. In particular, the recently discovered coronavirus SARS-CoV-2 primarily invades the pulmonary alveolar epithelial cells and may result in acute respiratory distress syndrome and occasionally into multi-organ failure. SARS-CoV-2 increases the risk of death and infection severity in COPD [76]. It has been hypothesized that the increased risk can be associated to cigarette smoke, the leading cause of COPD, which upregulates angiotensin converting enzyme 2 (ACE2), one of the most common SARS CoV-2 receptors in human lungs, most likely favouring viral dissemination [77]. However, the effects of cigarette smoke still controversial, as recent meta-analyses have questioned the relationship between SARS CoV-2 and smoking habit [78,79].

Viruses can alter the host environment favouring a secondary bacterial infection. Patients with viral/bacterial coinfection experience a strong limitation of respiratory functions and longer hospital stay [80]. Coinfection of COPD airways by viruses and bacteria is able to stimulate a higher production of proinflammatory cytokines (e.g. IL-6) and inflammatory biomarkers like C-reactive protein and procalcitonin [81] with respect to separate infection. Bacterial and viral coinfection was found in a relatively high percentage of patients by Wilkinson et al. [37,43] and Papi et al. [37,43]. They found viruses in 41% and 48%, respectively, of the exacerbated COPD patients, and viral and bacterial coinfection in 29% and 25%, respectively, of samples.

Mallia et al. [72] observed a subsequent bacterial infection in 60% of the patients infected with RV. Similar results were observed by Molyneaux et al. [82] who reported a bacteria overgrowth, especially *H. influenzae*, which persisted for more than a month after experimental infection with RV in COPD patients. Influenza virus is extremely efficient in favouring a bacterial overgrowth and a subsequent bacterial infection in normal subjects and COPD patients. In animal models, influenza viruses favour the secondary infection from pathogenic bacteria such as *S. aureus*, *S. pneumoniae* and *H. influenzae* [83]. The analysis of lung specimens from people deceased from pandemic influenza in 1918 and 2009 shows that the main cause of death was a subsequent bacterial pneumonia [84]. Influenza has a more severe course in COPD patients by increasing the risk of death [84] and hospital admission [85] with respect to normal subjects. The vaccination against influenza reduces the risk of exacerbation in COPD [86].

The ability of respiratory viruses to favour a secondary bacterial infection relies on several factors. Viruses like influenza and RSV synthesize proteins with immunosuppressive activity. Influenza non-structural protein 1 lowers the activity of caspase-1 system [87], which is pivotal against a bacterial infection [88]. Influenza virus cytotoxin PB1-F2 enhances the mortality due to *S. aureus* and *S. pneumonia* infections [89], and RSV G protein hampers the *in vitro* production of type I IFN and TNF-α by the immune system [90]. Non-structural 1 and 2 proteins of RSV reduce the production of interferon (IFN) alpha and beta, and alter the activity of the adaptive immune system.

**Figure 3.**
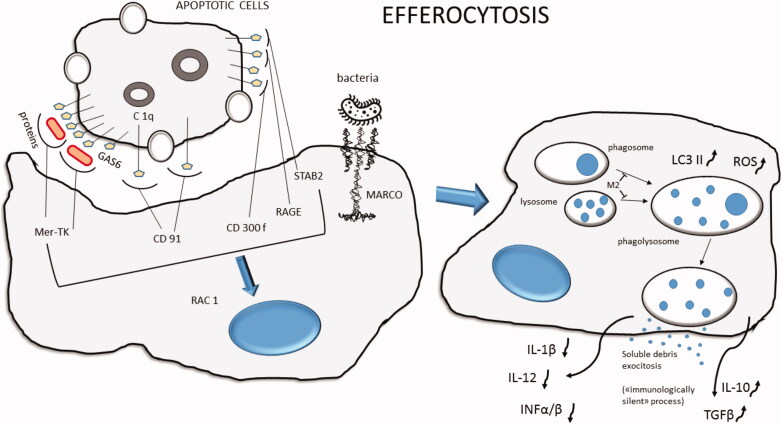
Schematic representation of efferocytosis for apoptotic cells or bacteria. The phagocytized material is transported inside the cell cytoplasm and processed as phagosome. Then, it merges with one or more lysosomes to produce a phagolysosome complex, where the action of enzymes and ROS degrades phagocytized cells. Viral derived M2 protein may reduce the phagolysosome formation. At the end of the process, debris will be exocyted from the cell. The process is considered “immunologically silent” with a low level of pro-inflammatory cytokine production like IL-1 β, IL-12, IFN α/β. MARCO: macrophage receptor with collagenous structure; Stab2: stabilin receptor-2; RAGE: receptor for advanced glycation end products; RAC1: Ras-related C3 botulinum toxin substrate 1; Mer-TK: proto-oncogene tyrosine-protein kinase MER; ROS: reactive oxygen species; LC3 II: microtubule-associated protein 1A/1B-light chain 3 phosphatidylethanolamine conjugate.

Respiratory viruses can use for their advantage some cellular mechanisms of cell death like apoptosis, autophagy, necroptosis and pyroptosis (Figures 2 and 3). One of the functions of these mechanisms is to help the immune system in stimulating the production of proinflammatory cytokines, and to present viral and bacterial components to T cells for an adaptive immune response [91]. Picornaviruses, like the human RV, use the double membrane compartments formed during the autophagic process as scaffold for their replication [92]. In infected cells, RSV inhibits apoptosis by reducing the level of P53, and induces autophagy, necroptosis and pyroptosis activating NF-κB activity [93,94]. Influenza viruses are strong apoptosis inducers in early phase of infection but induce pyroptosis during the late phase of the infection [95] (Figure 2). Influenza haemagglutinin and M2 protein both stimulate autophagy, while the M2 protein blocks the lysosome autophagosome fusion process [96] (Figure 3). In this way, the virus reduces the possibility for cells to degrade its components and uses autophagosome to optimize the replication [97].

Viral infections may directly damage epithelial cells by killing them or altering their barrier function against external agents, which can facilitate the subsequent bacterial colonization [98]. Mucus overproduction during a viral infection, associated with an impairment of ciliated cells, can hamper its removal from the airways therefore worsening airways’ obstruction in COPD patients [99]. The virus-induced production of MUC5AC, a mucin glycoprotein component of the airway mucus, seems to increase in COPD with respect to normal subjects [100]. The MUC5AC expression positively correlated with bronchial inflammation, virus load, secondary bacterial infections and clinical degree of COPD exacerbation. MUC5AC could exert its proinflammatory effect by stimulating epithelial cells to release extracellular adenosine triphosphate [101].

Respiratory viruses can increase the ability of some pathogens like *S. aureus*, *S. pneumoniae* and *H. influenzae* to adhere to lung epithelial cells by favouring the expression of adhesion molecules like fibronectin, ICAM-1 and platelet activating factor receptor [102]. Other proteins like influenza haemagglutinin and the RSV attachment glycoprotein (G) induced by respiratory viruses on the surface of the infected cells have a role in facilitating the adherence and internalization of pathogenic bacteria [103]. Moreover, respiratory viruses modulate the expression of some host O‐linked glycoproteins and sialic acid, mucin and fibrinogen‐like residues on the surface of MDCK cells, which increased bacterial adherence and/or internalization [104]. Viral infections stimulate an immune Th1-type response, which helps to increase IL-12, IFN-γ, IL-2 and TNF-α levels [105]. This polarized response can alter the equilibrium between IL-17 and IL-10 secretion, and increases susceptibility to subsequent bacterial infection [106].

Reduction of IL-17 secretion has been reported to enhance susceptibility to bacterial infections. In human lung macrophages and in a mice model with bacterial pneumonia after influenza virus infection, Podsiad et al. [107] reported a reduction of bacterial clearance efficiency due to a reduced production of IL-17. It was mediated by an increased expression of miR-155, which is a non-coding microRNA (miRNA) induced by different cytokines like IFN gamma, and acts post-transcriptionally as regulator of gene expression. An increased release of IL-17C was observed in COPD patients bronchial epithelial cells exposed to HRV and bacteria, which could lead to an increased neutrophil recruitment and inflammation of airways [108]. It was also reported that in mice model, the secretion of IL-10 induced by the influenza virus reduced the clearance of *S. pneumoniae* by NK cells [109], and that the use of an antibody against IL-10 reduced the mortality to a secondary bacterial infection [110].

In macrophages, the cytokine secretion induced by viral infections raises the expression of CD-200 receptor ligation antigen and lowers the expression of the macrophage receptor with collagenous structure [105]. It suppresses the activation of alveolar macrophages (AMs) [105], induces their apoptosis, impairs their cytokine production and the ability to phagocytize after challenging with bacterial products [111–113]. The dysfunction of macrophages, cells that orchestrate the immune response to pathogens, alters the activation and recruitment of the other immune system cells like neutrophils [111].

In mice, the IFNs released because of a viral infection [114,115], and the reduced production of IL-23 by DCs cause increased susceptibility to secondary bacterial infection [115]. NS1 and NS2 RSV proteins alter directly the maturation of human DCs reducing the effectiveness of the immune response [116]. Influenza viruses are able to inhibit neutrophil function [117], stimulating the production of IFN and IL-10 or directly infecting them [118]. Interestingly, Mallia et al. [72] suggested that the increased susceptibility to bacterial infection in COPD relies upon the ability of HRV-induced neutrophils elastase to cleave and reduce in sputum the levels of elafin and serine leukocyte peptidase inhibitor, neutrophilic peptides with antimicrobial activity. Influenza virus and RSV also stimulate neutrophil extracellular traps (NETs) secretion with a reduced antimicrobial activity [119], which becomes ineffective in capturing bacteria, but that could contribute to worsen the inflammation and the tissue damage in the airways.

Some authors have observed in mice a reduced cytotoxic activity and cytokine production of NK cells infected [120] by influenza virus and other respiratory viruses [121]. The impaired function of NK cells, which have a pivotal role in the control of viral infections [122] and organize an efficient immune response, could contribute to increase the susceptibility to infections from bacteria such as *S. aureus* [123]. Despite the fact that the majority of these studies are performed *in vitro* or in mice, it seems that viruses have the ability to cause a secondary bacterial infection worsening the outcome and rising the level of pro-inflammatory mediators in the airways.

## Altered immune response to bacteria and viruses in COPD

### Macrophages, dendritic cells, neutrophils and lymphocytes

Macrophages may play an important role in orchestrating the inflammatory process in COPD through the release of pro-inflammatory mediators including proteases, cytokines, chemokines and oxidative stress-related molecules [1,124]. In COPD, those cells showed reduced phagocytic activity, which may increase the persistence of the inflammatory process and impair the clearance of bacterial and viral pathogens [8,125]. CD68+ cells (macrophages) are increased in the bronchial mucosa of mild/moderate and severe/very severe COPD patients compared to controls [1,2]. It has been reported that numbers and percentages of CD163+, CD204+ and CD206+ AMs, belonging to M2-type macrophages secreting more MMP9, are increased in severe/very severe COPD compared to mild disease and controls [126], but their ability to phagocytize *H. influenza* [127,128], *M. catarrhalis* [127,128] and *S. pneumoniae* [127] is reduced. This could be explained by observing that exposure to cigarette smoke and air pollutants impairs the phagocytic AM activity [129]. Macrophage efferocytosis (Figure 3), a function that clears apoptotic neutrophils and structural cells and in doing so prevents the release of proinflammatory intracellular molecules, is also impaired in COPD patients especially after exposure to cigarette smoke [130–132]. Another impairing mechanism of the AM phagocytosis/efferocytosis is the alteration in kinase signalling and the decrease in ROS intracellular production [100] (Figure 3). These alterations in macrophages’ function could contribute to increase the bacterial load and modify the microbiota composition in the airways of stable COPD. Moreover, the impairment in macrophages function in stable COPD could be further worsened during viral infections [105,112], leading to a bacterial overgrowth and favouring bacterial exacerbations.

DCs are potent antigen-presenting cells with a key role in the regulation of immune responses. They also play a role in activating memory T-cell responses [133]. DCs are mainly divided into myeloid and plasmacytoid, which partially differ in their function and anatomic location [134]. Mature CD83+ DCs are decreased in sputum of stable COPD patients compared to control groups [135]. In the bronchial epithelium and lamina propria, a reduction of DCs is also reported in COPD patients compared to controls [136], and the chemokine receptor CCR5, involved in the uptake of microbial antigens and expressed on myeloid DCs, is reduced [137]. These data support the view of an impaired DC function in COPD.

In COPD patients’ airways, Garcia-Valero et al. [138] observed a reduced IFN-beta expression, a cytokine produced by plasmacytoid DCs but also by interstitial macrophages and epithelial cells. This reduced production could explain the augmented susceptibility of these patients to acute viral infections.

More recently, however, increased NK cytotoxicity against lung epithelial cells has been reported, primarily mediated by lung DC priming via IL-15 and IL-15Rα [139]. Furthermore, at multi-colour flow cytometry, circulating plasmacytoid DCs show an enhanced activation profile in patients with COPD contributing to an increase of IFNγ and IL-17-producing CD8^+^ T cells [140]. Viruses and bacteria alter the functioning of DC cells aiming at evading the immune response system [115,116,141].

The number of neutrophils is increased in the sputum, BAL, bronchial biopsies and peripheral airways of COPD patients compared to controls [1,2,7]. In parallel, molecules stimulating the neutrophil migration and activation are also increased. Macrophage inflammatory protein-1 (MIP-1α) in the bronchial epithelium is increased in severe COPD with respect to mild COPD and control smokers [2]. The analysis of pro-neutrophilic chemokines showed higher levels of RANTES (CCL5) and NAP-2 (CXCL7) in bronchial biopsies of severe stable COPD compared to control non-smokers [2,142]. It was also present an increased neutrophilic expression of CD44, involved in the increased neutrophilic adhesiveness to the extracellular matrix, or an increased neutrophilic expression of the activating receptor CD11b, particularly in the neutrophils from severe COPD compared to control subjects [8]. These characteristics may contribute to an increased permanence of these cells in the bronchial tissue of severe COPD patients. Interestingly, in COPD patients infected with RV during the exacerbation, Mallia et al. [143] observed an increased lung recruitment of neutrophils that express CD11b. Moreover, bacteria and viruses colonizing COPD airways in stable state stimulate the secretion of pro-inflammatory cytokines [14,15,50]. This could favour the recruitment of neutrophils in the airways of COPD patients even in stable state. The recently described release of NETs is an important immune mechanism capable of capturing pathogens [144]. An excess of NET formation damages the epithelium and may lead to lung tissue damage; it has also been reported in patients with COPD [145]. The NETs secretion is increased during respiratory virus infection, and this could bring about an adjunctive tissue damage [119]. Evasion of NETs by pathogens may increase resistance to the microbicidal NETs’ components, increasing the risk of airway infections in COPD [146]. More detailed studies are needed to better define the role of NET formation and its evasion in different clinical conditions of COPD patients [145].

Lymphocytes, mainly CD8^+^, are isolated in bronchial biopsies of COPD patients with mild/moderate disease, while their number decreases with the progression of the disease [2]. Lymphocytes sampled from peripheral blood of COPD show a higher tendency to undergo apoptosis when compared with those collected from patients without COPD [147]. More recently, it has been reported that in the airways of COPD patients, with respect to normal subjects, there is a higher percentage of T cells lymphocytes with reduced ability to degranulate cytotoxic proteins. The latter are able to induce apoptosis in tumoural or virus-infected cells, showing signals of functional exhaustion due to chronic antigenic stimulation as the expression of the programmed cell death protein (PD)-1. At the same time, the proliferation of functionally suppressive regulatory T cells observed in COPD patients’ airways further contributes to reduction of the activity of CD8^+^ cells in response to bacteria and viruses [148].

Similar findings have been reported in blood of COPD frequent exacerbators. Geerdink et al. [149] observed a reduction in the number of CD4^+^ central memory T cells and CD8^+^ activated effector memory T cells with respect to COPD patient infrequent exacerbators. The author hypothesized that the alteration in CD4^+^ and CD8^+^ lymphocytes population in COPD frequent exacerbators is due to the chronic stimulation by a high load of persistent antigens from bacteria and viruses present in their airways. Following the authors, the reduction of this lymphocytes population that has a pivotal role in orchestrating an immune response against pathogens already recognized from the immune system favour the occurrence of frequent exacerbations.

The alterations of the inflammatory cells in patients that appear more susceptible to exacerbations irrespective of disease severity should be better understood [55]. It would allow the development of personalized tailored treatments based on the inflammatory response. Moreover, how the immune system alterations affect the response to various pathogens like bacteria and viruses should be deeply addressed.

## Conclusions

Recent studies on COPD patients are clarifying the role of the microbiota in inflammation and lung deterioration. Those microorganisms, together with chronic inhalation of cigarette smoke and oxidative stress markers can influence the responses of the innate and adaptive immune system in the lung of COPD patients. Promising data have been published showing the close relationship between viruses and bacterial colonization, and the consequences of the imbalance of these components on the inflammatory state of the COPD lung. The complex actions involving immune trigger cells, which activate innate and cell-mediated inflammatory responses, interacting with external bacterial/viral and oxidant challenges, could be responsible for the clinical consequences of irreversible airflow limitation, lung remodelling and emphysema that these patients develop. Understanding the dynamics of these inflammatory and structural changes related to bacterial/viral colonization in different clinical conditions, under different treatments and in different phenotypes (e.g. prevalent airways disease vs. prevalent emphysema, frequent exacerbators vs. non-frequent exacerbators, treated with steroids, antibiotics or beta2 agonists) of COPD patients will improve our knowledge on the pathologic and molecular mechanisms underlying COPD.

## Data Availability

Data sharing is not applicable to this article as no new data were created or analysed in this study.
